# Increased HSF1 expression predicts shorter disease-specific survival of prostate cancer patients following radical prostatectomy

**DOI:** 10.18632/oncotarget.25756

**Published:** 2018-07-27

**Authors:** Johanna K. Björk, Ilmari Ahonen, Tuomas Mirtti, Andrew Erickson, Antti Rannikko, Anna Bützow, Stig Nordling, Johan Lundin, Mikael Lundin, Lea Sistonen, Matthias Nees, Malin Åkerfelt

**Affiliations:** ^1^ Institute of Biomedicine, University of Turku, Turku, Finland; ^2^ Department of Mathematics and Statistics, University of Turku, Turku, Finland; ^3^ Department of Pathology, Medicum, University of Helsinki, Helsinki, Finland; ^4^ Institute for Molecular Medicine Finland, University of Helsinki, Helsinki, Finland; ^5^ Department of Pathology, HUSLAB, Helsinki University Hospital, Helsinki, Finland; ^6^ Department of Urology, University of Helsinki and Helsinki University Hospital, Helsinki, Finland; ^7^ Faculty of Science and Engineering, Åbo Akademi University, Turku, Finland; ^8^ Centre for Biotechnology, University of Turku and Åbo Akademi University, Turku, Finland

**Keywords:** biomarker, heat shock factor, predictive marker, prostate cancer, tissue microarray (TMA)

## Abstract

Prostate cancer is a highly heterogeneous disease and the clinical outcome is varying. While current prognostic tools are regarded insufficient, there is a critical need for markers that would aid prognostication and patient risk-stratification. Heat shock transcription factor 1 (HSF1) is crucial for cellular homeostasis, but also a driver of oncogenesis. The clinical relevance of HSF1 in prostate cancer is, however, unknown. Here, we identified HSF1 as a potential biomarker in mRNA expression datasets on prostate cancer. Clinical validation was performed on tissue microarrays from independent cohorts: one constructed from radical prostatectomies from 478 patients with long term follow-up, and another comprising of regionally advanced to distant metastatic samples. Associations with clinical variables and disease outcomes were investigated. Increased nuclear HSF1 expression correlated with disease advancement and aggressiveness and was, independently from established clinicopathological variables, predictive of both early initiation of secondary therapy and poor disease-specific survival. In a joint model with the clinical Cancer of the Prostate Risk Assessment post-Surgical (CAPRA-S) score, nuclear HSF1 remained a predictive factor of shortened disease-specific survival. The results suggest that nuclear HSF1 expression could serve as a novel prognostic marker for patient risk-stratification on disease progression and survival after radical prostatectomy.

## INTRODUCTION

Prostate cancer is one of the most commonly diagnosed male cancer in Western countries, and worldwide approximately one million new prostate cancer cases are diagnosed each year [1]. The clinical course is highly variable; although primary prostate cancer often remains indolent, a considerable proportion progresses into castration-resistant prostate cancer. Advanced prostate cancer can rapidly metastasize both locally to the lymph nodes and distantly to bone, central nervous system, lung or other organs, and at this stage, despite therapeutic advances, there are still no curative treatments available and the metastasizing phenotype is lethal.

For localized prostate cancer a common treatment is radical prostatectomy. The most widely used tool to evaluate prognosis after primary treatment is Gleason grading, which is based on the glandular pattern of the tumor. However, accurate risk-stratification throughout the whole range of patients remains difficult, particularly for intermediate Gleason score tumors that often represent the vast majority of patients [2–4]. In addition, tumors with similar histological patterns can exhibit different clinical outcomes [5, 6]. Although Gleason grading was recently updated to a grade group system [7], and is being used in combination with other established parameters, foremost tumor stage and prostate-specific antigen (PSA), current prognostication is insufficient and accurate risk-stratification remains difficult. Additional information that allows more detailed and precise stratification of patients into distinct prognostic groups would be valuable. Thus, novel biomarkers for reliably assessing individual patient's risk of disease progression and outcome are highly needed.

Heat shock factor 1 (HSF1) is a ubiquitously expressed transcription factor that is crucial for cellular homeostasis and protection against protein damaging stress via the evolutionary conserved heat shock response. HSF1 also plays a vital role in tumor biology e.g. by promoting proliferation and survival upon oncogenic stimuli. Absence of HSF1 reduces proliferation and survival of human cancer cell lines, and protects mice from mutation- or carcinogen-driven tumors [8–13]. The tumorigenic property of HSF1 stems from activation of distinct transcriptional programs, including oncogenic support processes such as cell-cycle regulation, metabolism, adhesion, and translation, in both cancer cells and the tumor stroma. This demonstrates both cell-autonomous and non-cell-autonomous capabilities of HSF1 in orchestrating malignancy [14, 15]. Clinical relevance for HSF1 was demonstrated in breast, lung, and hepatocellular carcinoma where high mRNA and/or protein expression correlates with poor prognosis [14–18]. Although we have demonstrated that HSF1 promotes invasion in prostate cancer cell lines [19] and elevated expression has been detected in cancer cell lines and tumors [14, 19, 20], evidence for clinical significance of HSF1 in prostate cancer has not been demonstrated.

Here, we explore the prognostic value of HSF1 in prostate cancer by analyzing independent mRNA gene expression datasets and two separate, large prostate cancer patient tissue microarray (TMA) cohorts: one radical prostatectomy cohort with extensive clinical information and long term follow-up (15.7 years) and a second separate cohort comprising of regionally advanced to distant metastatic tumors. Associations with clinical variables and disease outcomes were investigated using proportional hazards regression (univariate, multivariate and LASSO-penalized Cox), binary decision tree model, Kaplan–Meier estimates, and log-rank tests. We hypothesized that the expression status of HSF1 may be associated with progression and aggressiveness of prostate cancer and that HSF1 can be utilized for outcome prognostications of patients who have undergone radical prostatectomy.

## RESULTS

### *HSF1* mRNA is overexpressed in prostate cancer

**Figure 1:**
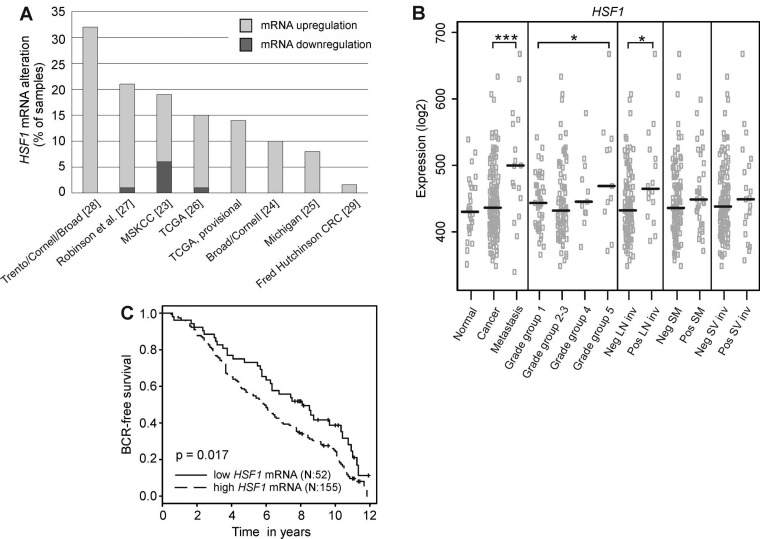
Analysis of *HSF1* mRNA in large-scale transcriptomics datasets (**A**) Expression status of *HSF1* in all publicly available clinical prostate cancer studies containing mRNA expression data in the cBioPortal (http://www.cbioportal.org) shown as percentages of samples with altered mRNA expression in the respective study. z-score ± 2. Numbers in parentheses refer to the respective reference. TCGA, provisional: data generated by the TCGA Research Network: http://cancergenome.nih.gov/. (**B**) Analysis of *HSF1* mRNA expression in a clinical prostate cancer dataset comprising of 216 samples [23] showing that *HSF1* expression is significantly increased in metastases, advanced prostate cancer (grade group 5), and lymph node invasion. The black line represents the median. Statistical significance was calculated using Mann-Whitney test. ^*^P-values < 0.05; ^***^P-values < 0.001. SM: positive surgical margins; SV inv: seminal vesicle invasion. (**C**) Kaplan–Meier estimates of biochemical recurrence (BCR)-free survival in *HSF1* mRNA expression groups, comparing 25% low-level expressing *versus* 75% high-level expressing prostate cancer tumors in the MSKCC dataset [23].

Since HSF1 has been demonstrated to be a strong promoter of oncogenesis, we hypothesized that HSF1 would hold clinical significance in prostate cancer. Previously, we have noted elevated levels of HSF1 mRNA when comparing human luminal prostate cancer cell lines to basal, benign prostate epithelial cell lines [19]. Thus, we first performed a large-scale analysis of *HSF1* mRNA expression using all clinical prostate cancer datasets in cBioPortal (http://www.cbioportal.org/public-portal) [21, 22] available at the time of analysis. This revealed high expression of *HSF1* mRNA in tumor samples across all datasets when compared to matched normal samples (Figure 1A) [23–29]. In-depth analyses on the transcriptomics dataset from MSKCC [23], containing comprehensive prostate cancer profiles of 216 clinical samples from both primary tumors and metastases were then performed. Statistically significantly elevated *HSF1* levels were found to be associated with high grade group prostate cancer, positive lymph node status and metastasis (Figure 1B), all signs of progressing disease. Next, the association between mRNA expression and biochemical recurrence (BCR) was analyzed by stratifying the patients into high *versus* low *HSF1* expressing groups. Interestingly, high *HSF1* mRNA levels were associated with poor BCR-free patient survival (*p* = 0.017; Figure 1C). These results imply clinical significance for HSF1 in advanced prostate cancer, and justify an in-depth analysis of HSF1 protein expression in large prostate cancer cohorts.

### Increased HSF1 protein expression corresponds to advancement of prostate cancer

**Figure 2:**
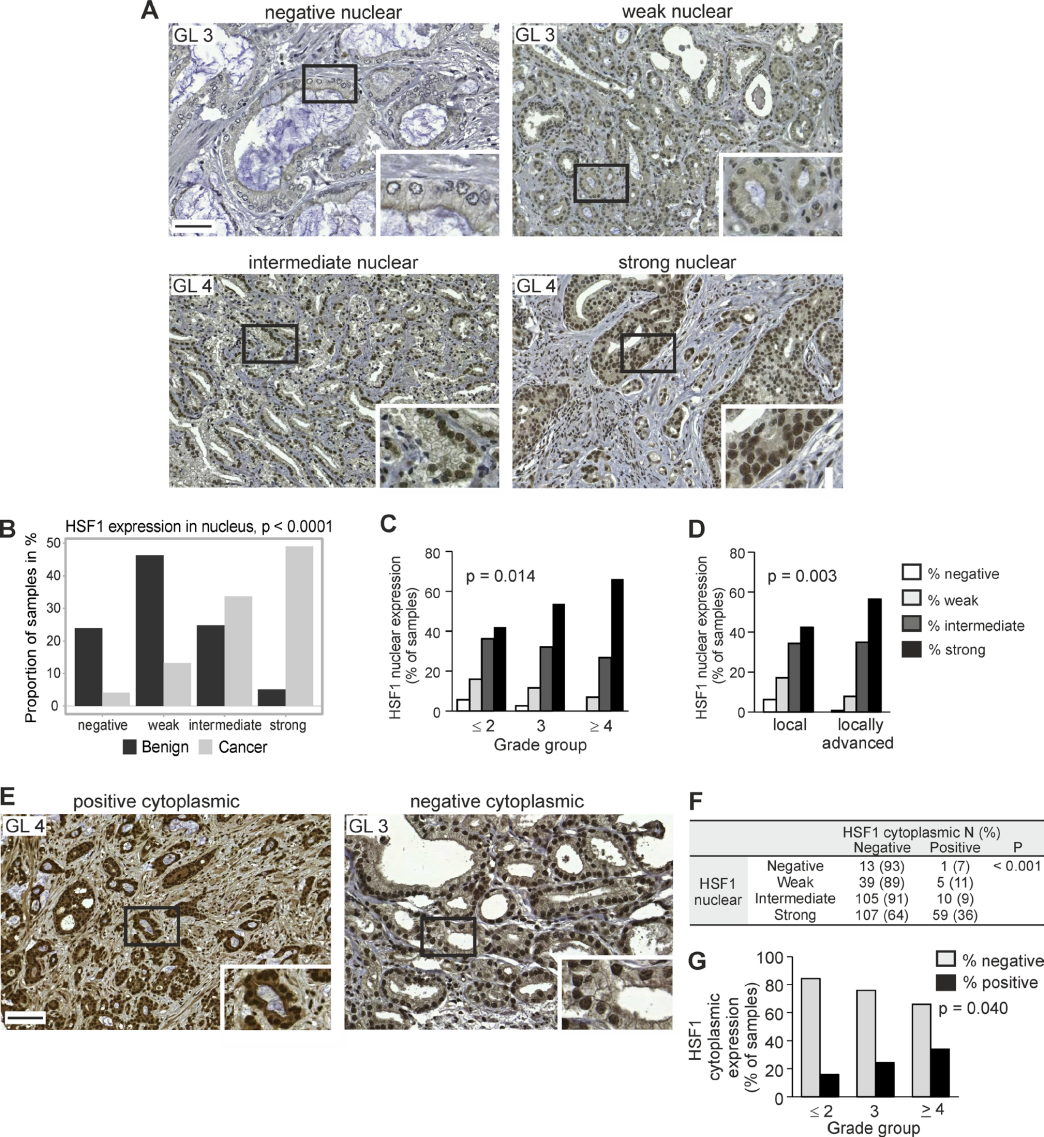
Increased HSF1 protein expression corresponds to the progression of prostate cancer (**A**) IHC staining of nuclear HSF1 protein on TMAs containing Gleason grade pattern 3–5 cores. Representative images are shown for negative (score 1), weak (score 2), intermediate (score 3), and strong (score 4) HSF1 expression. Scale bar represents 100 μm. Blow-up images in the insets. GL, Gleason grade. (**B**) Nuclear HSF1 protein staining status (negative, weak, intermediate, strong) in benign/normal and malignant prostate cancer biopsies. (**C**) Association between nuclear HSF1 protein staining status and grade group (GG), demonstrating that increased expression correlates with high grade group tumors. (**D**) Comparison of samples from local (pT2) and locally advanced (≥pT3) disease showing statistically significantly higher levels of nuclear HSF1 in tumors that have spread outside the prostate. (**E**) IHC staining on TMAs, showing representative images with both nuclear and cytoplasmic positive HSF1 expression (left) and with positive nuclear expression but negative cytoplasmic HSF1 expression (right). Scale bar represents 100 μm. Blow-up images in the insets. (**F**) Cross-tabulation of nuclear and cytoplasmic HSF1 protein expression. *N* = number of patients. (**G**) Association between cytoplasmic HSF1 protein staining status and GG.

We pursued the clinical relevance of differential expression of HSF1 by immunohistochemistry (IHC) [30] on a large primary prostate cancer cohort of radical prostatectomy samples, collected into TMA I (Supplementary Table 1) [31, 32]. In the final analysis, 368 patients had comprehensive clinical data and representative tissues in the TMA for analysis of HSF1. From each patient, three cores were obtained from the cancer areas and one core from an adjacent benign/normal area. HSF1 was mainly detected in the nucleus, and the signal was scored as negative, weak, intermediate, or strong (score 1–4, respectively; Figure 2A; Supplementary Figure 1). Intriguingly, a clear majority (83%) of the prostate cancer cores showed intermediate to strong HSF1 expression, while 70% of the benign cores showed negative or weak HSF1 expression (Figure 2B). The association between HSF1 staining intensities and commonly used clinicopathological variables was further investigated by cross-tabulation and summary statistics (Table 1). This revealed that enhanced nuclear HSF1 expression was significantly associated with higher grade groups (*p* = 0.014), positive lymph node status (*p* = 0.017), and locally advanced (≥pT3) compared to organ-confined (pT2) disease (*p* = 0.003) (Figure 2C and 2D; Table 1). These results are in agreement with the increased *HSF1* mRNA levels detected in advanced prostate cancer and local metastases (Figure 1B).

**Table 1:** Association between the level of nuclear and cytoplasmic HSF1 expression and tumor characteristics in TMA I

Tumor characteristics		Nuclear HSF1 score			Cytoplasmic HSF1 score		
		1–3	4	*P*^a^	Negative	Positive	*P*^a^
		*n* (%)	*n* (%)		*n* (%)	*n* (%)	
Grade group	1 (4–6)	41 (58.6)	29 (41.4)	0.033	56 (81.2)	13 (18.8)	0.050
(Gleason score)	2 (3 + 4 = 7)	50 (57.5)	37 (42.5)		77 (86.5)	12 (13.5)	
	3 (4 + 3 = 7)	52 (46.4)	60 (53.6)		85 (75.9)	27 (24.1)	
	4 (8)	14 (32.6)	29 (67.4)		27 (64.3)	15 (35.7)	
	5 (9–10)	5 (38.5)	8 (61.5)		10 (71.4)	4 (28.6)	
pT	2	101 (57.7)	74 (42.3)	0.003	143 (81.7)	33 (18.3)	0.16
	≥3	55 (43.7)	71 (56.3)		95 (74.8)	32 (25.2)	
Positive surgical	Yes	29 (51.8)	27 (48.2)	1.00	50 (86.2)	8 (13.8)	0.12
margin	No	143 (50.9)	138 (49.1)		213 (76.1)	67 (23.9)	
Lymph node status	Benign Metastasis	172 (52.6) 1 (11.1)	155 (47.4) 8 (88.9)	0.017	258 (78.7) 7 (77.8)	70 (21.3) 2 (22.2)	1.00
PSA μg/L^b^ (SD)		15.3 (±14.7)	13.5 (±12.3)	0.13	15.0 (±14.2)	12.1 (±10.7)	0.047

^a^*P*-values are calculated using the Fisher´s exact test for categorical variables and Wilcoxon test for continuous variables.

^b^PSA is preoperative and values are mean.

Abbreviations: SD, standard deviation; PSA, prostate-specific antigen.

Interestingly, in approximately 20% of the prostate cancer samples with HSF1 expression status, HSF1 was also detected in the cytoplasm, where it was scored as negative (no staining) or positive (Figure 2E; Supplementary Table 1; Supplementary Figure 2). The incidence of positive cytoplasmic staining increased with higher nuclear HSF1 expression score and grade group (Figure 2F–2G).

### Strong HSF1 expression predicts risk of receiving secondary therapy

**Figure 3:**
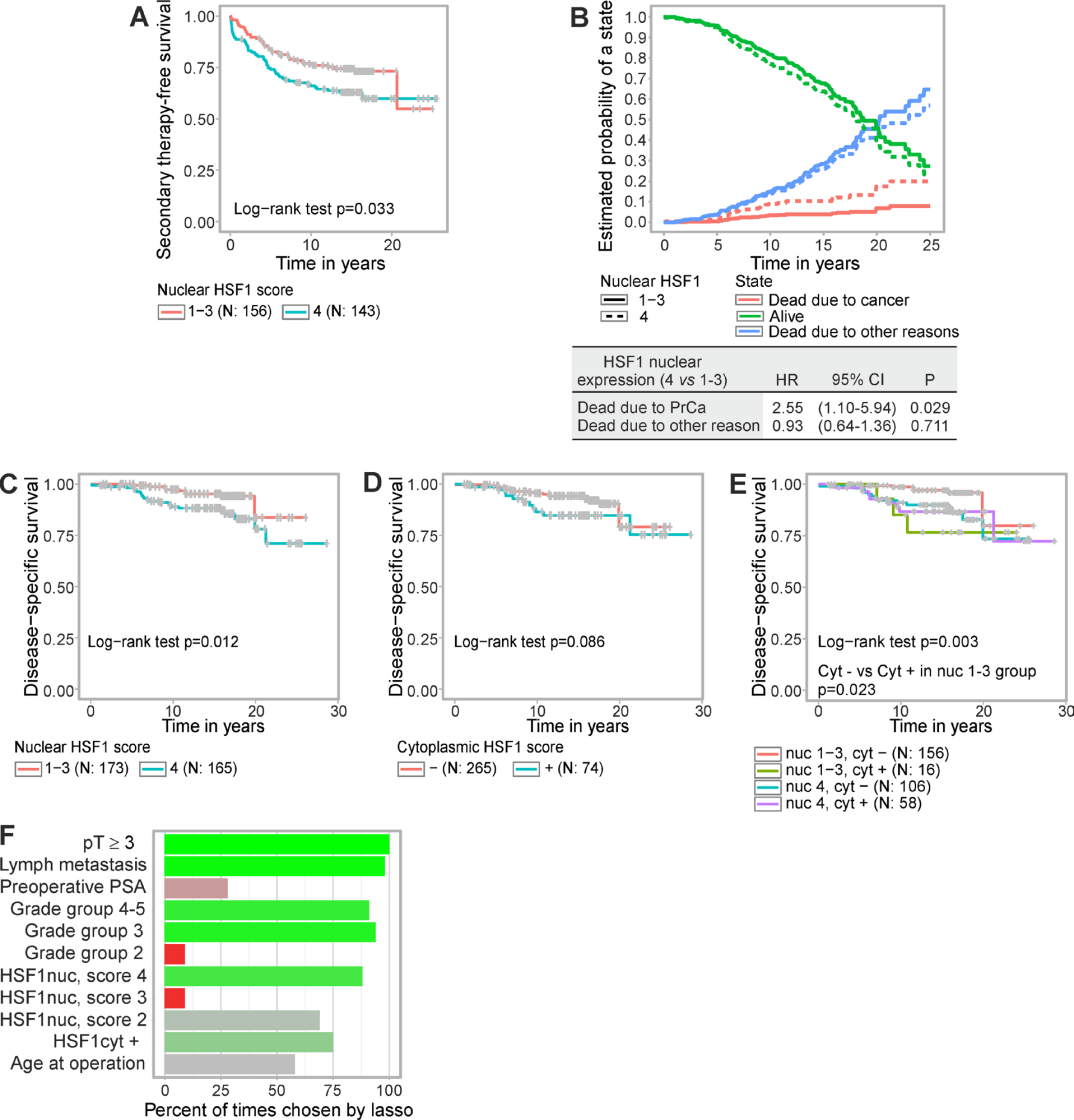
Elevated HSF1 expression predicts shorter time to secondary therapy and poorer disease-specific survival after radical prostatectomy (**A**) Kaplan–Meier estimates of time to secondary therapy after radical prostatectomy showing low (score 1–3) *versus* strong (score 4) nuclear HSF1 staining, (**B**) Competing risk analysis where the probability to be in a specific state; alive (green), dead due to prostate cancer (red), and dead due to other reasons (blue), were estimated in relation to nuclear HSF1 expression, i.e. strong (score 4) *versus* low (score 1–3). (**C**–**E**) Kaplan–Meier estimates of disease-specific survival showing (C) strong (4) *versus* low (1–3) nuclear HSF1 staining, (D), negative or positive cytoplasmic HSF1 staining, or (E) combinations of nuclear and cytoplasmic HSF1 staining. (**F**) Variables chosen by the LASSO-regression model combined with multiple imputation and with disease-specific survival as the end point. The covariates were tumor extension (pT), lymph node status, log preoperative PSA, GG, nuclear HSF1 staining, cytoplasmic HSF1 staining, and age at operation. Bright green indicates prognostic value; bright red indicates equivocal prognostic value; shades of grey indicate that no conclusions can be drawn from these data. *N* = number of patients.

We assessed the connection between HSF1 expression status and the likelihood of receiving secondary therapy after radical prostatectomy. Treatment decisions for the patients were made by practicing urologists and reflect the clinical practice at the time. Analysis of the Kaplan–Meier estimates showed that strong nuclear HSF1 staining (score 4) was associated with shorter secondary therapy-free survival compared to low nuclear HSF1 staining (score 1–3) (Figure 3A, *p* = 0.033). Next, a univariate Cox regression model showed that strong nuclear HSF1 staining raised the risk of receiving secondary therapy with a hazard ratio (HR) of 1.56 (95% CI 1.03–2.35; *p* = 0.035) (Supplementary Table 2). When assessing the relationship between HSF1 and established clinical markers, a correlation matrix showed no marked dependencies (Supplementary Figure 3). In a multivariate Cox analysis that took established clinical markers into account, nuclear HSF1 staining remained an independent predictor of secondary therapy (HR 1.77; 95% CI 1.03–3.02; *p* = 0.037) (Supplementary Table 2).

### Enhanced HSF1 expression correlates with poor disease-specific survival

Next, we studied the relationship between HSF1 expression and prostate cancer survival. In order to assess the utility of HSF1 as a prognostic marker further a competing risk analysis was performed where the probability to die specifically from prostate cancer, to die from other reasons, or to stay alive were compared in relation to the expression status of nuclear HSF1 (Figure 3B). Interestingly, strong nuclear HSF1 expression clearly increased the likelihood to die from the disease (HR 2.55; 95% CI 1.10–5.94; *p* = 0.029). No evidence was found for HSF1 affecting the probability of dying for other reasons. To investigate the prognostic value of HSF1 in different patient groups and in relation to grade group on disease-specific survival, a survival decision tree was generated (Supplementary Figure 4A). The individual nuclear and cytoplasmic HSF1 scores as well as grade group were supplied as factors to the algorithm after which no further user interaction was required. Survival up to given time points in the patient groups derived by the tree is illustrated in Supplementary Figure 4B. The resulting tree revealed that while grade group remained the most informative prognostic factor (node 1), nuclear HSF1 expression status added to the survival prediction for advanced cancers (node 5; Supplementary Figure 4A). Simultaneously, the decision tree confirmed the division of nuclear HSF1 status used in analyses above, i.e. low (scores 1–3) *vs* strong (score 4) expression, as the most informative cut point (node 5). Cytoplasmic HSF1 enhanced the prognostic value when the grade group was low (≤ 2; node 2).

Kaplan–Meier graphs of disease-specific survival verified the findings by the tree model by showing that strong nuclear HSF1 expression predicted earlier patient death (*p* = 0.012; Figure 3C). At 10 years post-operation the likelihood of disease-specific survival was decreased by 8.4% (95% CI 2.8–14.0; Figure 3C). Disease-specific survival was not statistically significantly affected when stratifying patients by cytoplasmic HSF1 staining (Figure 3D). When combining nuclear and cytoplasmic HSF1 staining, patients with low nuclear expression (score 1–3) and negative cytoplasmic HSF1 showed the longest disease-specific survival time compared to the other groups (Figure 3E). Cytoplasmic HSF1 also added to the prognostic value of the low-risk group with nuclear HSF1 score 1–3 (Figure 3E, *p* = 0.023).

### HSF1 is a prognostic marker for prostate cancer-specific death

For prostate cancer-specific death, a univariate Cox proportional hazards model showed that strong nuclear HSF1 staining raised the risk with a HR of 2.62 (95% CI 1.21–5.67; *p* = 0.015; Table 2). When incorporating nuclear HSF1 together with grade group alone in a multivariate model, strong nuclear HSF1 remained an independent factor with a HR of 2.40 (95% CI 1.06–5.41; *p* = 0.035; Table 2). This should be compared to the HR of GG, which was 1.78 (95% CI 1.28–2.48; *p* = 0.001). Possibly due to the small number of events (4.9% of the patients were deceased due to prostate cancer), the association with disease-specific survival did not reach statistical significance if additional variables were included into the model (Supplementary Table 3).

**Table 2:** Univariate and multivariate Cox proportional hazards models on disease-specific survival in prostate cancers

Univariate	Disease-specific survival		
	HR	95% CI	*P*
HSF1 nuclear (score 4 vs 1–3)	2.62	1.21–5.67	0.015
HSF1 cytoplasmic (positive vs negative)	1.91	0.90–4.04	0.091
Grade group	1.88	1.38–2.56	<0.001
pT (≥T3 *vs* T2)	12.85	3.86–42.82	<0.001
PSA	1.03	1.01–1.05	0.011
Positive surgical margin	0.62	0.22–1.77	0.37
Lymph node metastasis	5.05	1.75–14.55	<0.001
**Multivariate^a^**	HR	95% CI	*P*
HSF1 nuclear (score 4 *vs* 1–3)	2.40	1.06–5.41	0.035
Grade group	1.78	1.28–2.48	0.001
**Multivariate + MI**	HR	95% CI	
CAPRA-S	1.27	1.11–1.46	
HSF1 nuclear	2.27	1.04–4.98	

^a^Number of patients, 324; number of events, 31.

Abbreviations: CI, confidence interval; HR, hazard ratio; MI, multiple imputation.

To widen the range of clinical variables examined together with HSF1, we utilized the least absolute shrinkage and selection operator (LASSO)-regression model in combination with multiple imputation to account for missing values in the covariates. Four variables; pT ≥ 3, metastatic lymph node status, grade group 3 and 4–5, and strong HSF1 nuclear staining (score 4) were independently chosen by LASSO in over 75% of the imputed datasets suggesting that these hold independent prognostic value for disease-specific survival (Figure 3F). Further investigations on HSF1's performance were assessed by the full clinical model CAPRA-S, which includes the variables PSA, GS in the radical prostatectomy specimen, surgical margin, seminal vesicle invasion, extracapsular extension, and lymph node invasion [33]. After demonstrating non-dependency between HSF1 and CAPRA-S in an analysis of variance in which the average CAPRA-S scores did not differ statistically significantly between the nuclear HSF1 groups (*F*-test *p* = 0.221), multivariable analysis combined with multiple imputation was performed. This revealed a HR of 2.27 (95% CI 1.04–4.98) for a combined model with nuclear HSF1 and HR 1.27 (95% CI 1.11–1.46) for CAPRA-S (Table 2). The average *p*-value from the imputed datasets in a subsequent likelihood-ratio test comparing the joint model and the CAPRA-S score alone was 0.032, indicating that HSF1 brings added prognostic value on top of the CAPRA-S score. Taken together the results demonstrate that nuclear HSF1 is an independent prognostic marker from the currently established variables for guiding treatment decisions, and could potentially be used in clinical practice.

### HSF1 is excessively expressed in advanced and metastatic tumors

**Figure 4:**
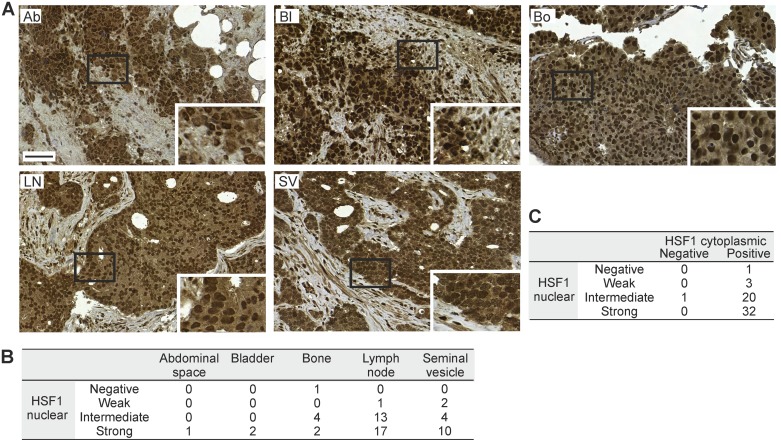
HSF1 protein expression in samples from regionally advanced to distant metastatic sites of prostate cancer (**A**) IHC stainings of HSF1 on TMA II containing samples from the abdominal space (Ab), bladder (Bl), bone (Bo), iliac lymph node (LN), and seminal vesicle (SV). Scale bar represents 100 μm. Blow-ups in the insets. (**B**) Distribution of nuclear HSF1 intensity at the different sites. (**C**) Cross-tabulation of the level of nuclear and cytoplasmic HSF1 expression in the samples. (B–C) The numbers designate patients.

Finally, to verify HSF1 stainings on TMAs and since HSF1 levels, both mRNA and protein, were found to increase with prostate cancer progression (Figure 1B; Figure 2C; Table 1), we performed IHC on a large cohort from advanced prostate cancers (TMA II). This independent TMA contained 103 cores from the seminal vesicles and from metastases in the abdominal space, bladder, bone and local lymph nodes from 57 patients. Representative images from the seminal vesicles and metastatic sites showed HSF1 expression in infiltrating tumor cells (Figure 4A). Strikingly, intermediate or strong HSF1 nuclear expression was detected in the vast majority (93%) of the advanced cancer cores, and all except one of the cores simultaneously showed positive cytoplasmic HSF1 staining (Figure 4B–4C). The excessive amounts of both nuclear and cytoplasmic HSF1, independently of the metastatic site, highlight HSF1 as a key factor in invasion and metastasis of prostate cancer.

## DISCUSSION

HSF1 is foremost known for mediating the heat shock response, a highly conserved mechanism that protects the cell from environmental and pathological proteotoxic damage [34]. In cancer, proteotoxic stress arises from various sources such as the heightened degree of aneuploidy, accumulation of mutated proteins and the harsh conditions of the tumor microenvironment [35, 36]. Not surprisingly, HSF1 expression has been found to be elevated in various cancer cell lines and cancer types and HSF1 identified as a driver of carcinogenesis [8, 10, 11]. It appears that cancer cells may hijack HSF1 functions and its transcriptional activity to promote survival, growth and metastatic propensity [13, 14]. While the oncogenic potential of HSF1 has been thoroughly demonstrated on a molecular level, its clinical significance has only begun to be revealed [14, 16]. We recently demonstrated that the absence of HSF1 renders prostate cancer cell lines in a non-differentiating acinar state, non-invasive, and prone to cell death in 3D organotypic cell culture. Likewise, in the chorioallantoic membrane *in vivo* model, tumors derived from prostate cancer cell lines showed reduced growth upon knock-down of HSF1 expression [19]. In this study a clinical significance of HSF1 in prostate cancer is demonstrated: increased HSF1 expression associates with disease progression and independently predicts initiation of secondary therapy and poor disease-specific survival of prostate cancer patients after radical prostatectomy. This suggests that HSF1 could serve as a novel prognostic marker in prostate cancer.

The prognostic value of HSF1 was here demonstrated using a comprehensive radical prostatectomy cohort with extensive follow-up time necessary for clinically relevant end points: initiation of secondary treatment and disease-specific survival. TMA I revealed that increased nuclear HSF1 expression is evident in malignant tissues and correlates with disease progression. Another independent TMA (TMA II), composed of prostate cancer in seminal vesicles or metastases, as well as the mining of publicly available mRNA datasets verified our findings of increased expression during disease advancement. In accordance, enhanced *HSF1* mRNA expression has been detected in breast and hepatocellular carcinoma and enhanced nuclear HSF1 protein expression in a wide range of malignancies including hepatocellular carcinoma, breast, cervical, lung, pancreas, colon, and mesenchymal tumors [14, 16–18].

The clinical course of prostate cancer is highly variable and more accurate risk-stratification of patients is needed for informed therapeutic decision-making. One particular problem is the selection of individuals that are likely to benefit from locoregional and systemic treatment following radical prostatectomy of intermediate and high-risk patients. Despite extensive research to uncover reliable biomarkers that provide improved sensitivity over current tools, only three biomarkers are currently approved for clinical use by the US Food and Drug Administration (FDA): PSA, the related prostate health index that combines different PSA forms, and prostate cancer antigen 3 (PCA3), a long non-coding RNA. A number of additional biomarkers, such as TMPRSS2-ERG gene fusion test and tests containing panels of markers, are offered as Clinical Laboratory Improvement Amendments-based laboratory developed tests. However, none of these has of yet been approved by the US FDA for clinical practice [37]. This study suggests that HSF1 is a driver of prostate cancer progression and could serve as an informative biomarker for stratifying patients after radical prostatectomy. Strong nuclear HSF1 expression was, independently from established clinicopathological markers, associated with shorter time to secondary therapy. Importantly, strong nuclear HSF1 expression also predicted poor disease-specific survival, demonstrating HSF1 as a determinant of lethal disease. Of note, the prognostic HR values derived from HSF1 nuclear scores were comparable to those derived from common established clinical markers such as GG. In addition, independence from the full clinical model CAPRA-S, as demonstrated by variance analysis, allowed a combined risk analysis after multiple imputation. Subsequent likelihood-ratio test demonstrated the value of nuclear HSF1 in the model for predicting disease-specific survival.

Although the size and follow-up time of the patient cohorts used in this study were substantial and enabled pertinent end point analyses, the relatively low count of lethal events reduced the statistical power in subgroup and multivariate models. For these reasons, a multivariate Cox analysis of disease-specific survival was of only limited use. However, by utilizing LASSO-penalization and applying multiple imputation, these shortages were addressed and the prognostic value of HSF1 shown. Specifically, the joint analysis with the established CAPRA-S score with subsequent likelihood-ratio test verified HSF1 as an independent and relevant marker. Prospective studies on extensive, independent cohorts are however warranted to estimate the magnitude of the effect.

This study is based on samples taken after radical prostatectomy or at an advanced stage. For subsequent IHC analyses, HSF1 status could be surveyed in biopsies and connected to disease outcome for a prognostic value on progression and disease-specific survival, possibly saving patients from unnecessary radical prostatectomy and aiding earlier treatment. Although IHC is an indispensable technique in pathology laboratories, varying staining quality can pose a problem. However, potential problems can be overcome by following published guidelines on standardization [38, 39]. These include the use of an antigen retrieval method that enables detection from formalin-fixed and paraffin-embedded surgical specimens, automated staining, and digital pathology combined with imaging analysis. Furthermore, thorough antibody testing, optimization, and validation, including the use of positive and negative controls, is a prerequisite. In order to use HSF1 IHC analyses as an established method in clinics in the future, a standard operating procedure protocol should be developed.

Apart from the prognostic value, our findings hold therapeutic potential. HSF1 has been considered a target for anti-cancer therapy due to the dependency of malignancies on this non-oncogene and its overexpression in many cancers [40]. Several small molecular inhibitors of HSF1 have been identified [40, 41], and a water-soluble pro-drug of the inhibitor triptolide is currently in phase I clinical trial for advanced gastrointestinal tumors (NTCT01927965) and has demonstrated pre-clinical activity against hepatocellular carcinoma, osteosarcoma, and ovarian and pancreatic cancer [42–44]. Our results on differential expression of HSF1 open up for therapeutic interventions also in prostate cancer.

In conclusion, this study enhances the understanding of prostate cancer progression by demonstrating that the levels of HSF1 increases as the disease advances and that HSF1 status predicts disease-specific survival. Taken together, the study demonstrates that characterizing HSF1 expression with straight-forward and robust antibody-based detection holds potential for use in clinical practice, i.e. for risk-stratification and outcome predictions of patients treated with radical prostatectomy.

## MATERIALS AND METHODS

### Study design

Reporting recommendations for tumor marker prognostic studies; REMARK [45] was followed throughout the study. The study design is outlined in Supplementary Figure 5.

### Bioinformatics of mRNA expression in clinical samples

Expression status of HSF1 was investigated in all clinical prostate cancer studies available at the time of analysis through the cBioPortal for Cancer Genomics (http://www.cbioportal.org/public-portal) [21, 22]. For in-depth analysis, a clinical transcriptome study (Memorial Sloan Kettering Cancer Centre, MSKCC) [23] was used, containing 216 prostate cancer samples and metastases with comprehensive profiles, of which 85 displayed complete mRNA, copy number, sequencing data, and based on Affymetrix Human Exon 1.0 ST Arrays and next-generation sequencing. Gene expression data from curated and normalized values were analyzed in GeneSapiens [46]. Normalized raw expression data of the MSKCC collection was extracted, median centered, and analyzed through an in-house HTML interface, REX, which houses a collection of relevant R-scripts for mining of data and plotting of observations. Associations of gene expression with clinical annotations (e.g. grade group, invasion status) were processed with R.

### Clinical prostate cancer samples for tissue microarrays

Prostate cancer specimens from two independent clinical cohorts were constructed into TMA I and II. For TMA I, samples were obtained from 478 patients treated by radical prostatectomy during the years 1982–1998 at Helsinki University Hospital, Finland. For the final analysis, 368 patients without neoadjuvant treatment and with comprehensive data, HSF1 expression status, and tissue material available were included. Clinical preoperative and follow-up information including overall and disease-specific mortality data was gathered from the Finnish Cancer Registry, and updated in November 2015. The age of the patients at diagnosis ranged between 45 and 76 years, and none had received adjuvant therapy before or immediately after surgery. The median post-surgery follow-up time was 15.7 years (Supplementary Table 1). For TMA II, 103 samples from regionally advanced and distant metastatic sites of 62 patients treated at Turku University Hospital, Finland between 1993 and 2008, were used [47]. In the final analysis, cores from 57 samples were representative for scoring, including 1 abdominal, 2 bladder, 7 bone, 31 lymph node metastases and 16 seminal vesicle infiltration samples. The median age of the patients was 64 years, and some had received hormonal treatments. For both TMAs, histopathological features were independently reviewed by pathologists using hematoxylin-eosin or Herovici's collagen stained slides. Sample and clinicopathological data usage was approved by the ethics committees of Hospital District of Helsinki or Uusimaa and Hospital District of Southwest Finland, and the National Authority for Welfare and Health in Finland according to national legislation. Use of the Finnish Cancer Registry's data was approved by the National Institute for Health and Welfare. Patient data was de-identified prior to analyses.

### Construction of the TMAs and immunohistochemistry

The TMAs were constructed using archival formalin-fixed, paraffin-embedded blocks as described in [32]. For TMA I, the blocks from each patient were drilled from different areas to account for tumor heterogeneity: two cores from the area containing the most dominant Gleason grade pattern, one core form the area containing the second most dominant Gleason pattern, and one core from an adjacent benign glandular area. TMA I, comprising prostate cancer prostatectomy samples, contained a total of 1758 cores. TMA II, comprising disseminated prostate cancer samples, contained a total of 105 cores from regional and distant sites. All cancer cores were scored individually, according to the Gleason grade pattern. Freshly cut 4 μM thick TMA sections were mounted on electrically charged glass slides (SuperFrost Plus, Menzel-Gläser, Braunschweig, Germany), and stained using Lab Vision^™^ PT Module and Autostainer 480 (Thermo Scientific, Waltham, MA, USA) with heat-induced *epitope* retrieval in TRIS buffer. The slides were incubated with rabbit polyclonal HSF1 antiserum (1:1000), generated and described previously [30]. Shortly, purified 65–70 kDa recombinant human HSF1 was used for immunization of rabbits. Specificity was verified by Western blotting using recombinant human HSF1 and human cell lysates [30]. For the TMA, HSF1 staining of normal, human urinary bladder tissue was used as a positive control, showing consistency with stainings in Protein Atlas (https://www.proteinatlas.org/ENSG00000185122-HSF1/tissue). Rabbit IgG staining of prostate cancer tissue was used as a negative antibody control (Supplementary Figure 1).

### Digitalization and scoring of TMA cores

The TMAs were analyzed largely as described by Björkman *et al*., 2012 [32]. The immunostained TMA sections were digitized with an automated whole slide scanner (Mirax Scan, Zeiss, Göttingen, Germany) and virtual slides were uploaded to a web server (http://fimm.webmicroscope.net). Reliable Gleason grading was possible for 1064 out of 1104 prostate cancer TMA I cores stained with HSF1 antibody. HSF1 expression was evaluated by a pathologist (TM) independently from the digitized slides without information on grade and clinicopathological data. In all analyses, the maximum HSF1 score of the available prostate cancer cores for each individual patient was used. The nuclear staining intensity was scored as: no staining (negative, score 1), weak (score 2), intermediate (score 3), or high (score 4), and the cytoplasmic staining was scored as: no staining (negative) or positive. To assess the reliability of the HSF1 measurements, the correlation structure of the scores was investigated (Supplementary Figure 6). In short, little or no dependency was found between scores from differing sources, while scores from the same source were moderately correlated (Spearman *r* = 0.57–0.66). This level of agreement can be considered satisfactory, however, low enough to justify multiple samples from each patient.

### Statistical analysis

Associations between categorical variables were investigated by cross-tabulation and Fisher's exact test. Differences in average values of continuous variables between factor groups were assessed with non-parametric Wilcoxon signed-rank test. Survival analyses were performed with Kaplan–Meier estimates and log-rank tests. HRs were obtained by fitting Cox proportional hazards regression models for single and multiple prognostic factors. Correlations between variables were estimated using the non-parametric Spearman's rank correlation.

A binary decision tree [48] was utilized to provide an automated approach to deriving useful prognostic groups. In contrast to the Cox models using only the main effects of the variables, this approach makes minimal assumptions on the structure of the data and is able to detect possible interactions between covariates and nonlinear dependencies. The tree was constructed by consecutively splitting the patients into groups at points that minimize the log-rank test *p*-value. The splitting point is chosen among all covariates supplied to the algorithm. The method can thus also be used for identifying useful prognostic factors.

Least absolute shrinkage and selection operator (LASSO)-penalized Cox regression [49] was used for picking the most relevant variables among a large set of prognostic factors. The method aims to set the HR related to unnecessary prognostic factors to exactly 1, thus negating their effect in the model. The method is controlled by a hyperparameter λ, whose value was chosen using 5-fold cross-validation [50].

Multiple imputation [51] was used to address the missing values of variables other than HSF1 expression in the data. The method imputes any missing data with reasonable guesses by taking into account the (non-missing) values in the other variables. The guesses are random, and thus to characterize the full uncertainty in the missing value estimation, the procedure is repeated multiple times. This results in a set of imputed datasets that differ in their imputed missing values. The subsequent analyses are then performed on each of the datasets and the results are combined for the final analysis using Rubin's rules [52].

Multivariate analyses with Cancer of the Prostate Risk Assessment post-Surgical (CAPRA-S) score were performed as detailed in [33]. In an analysis of variance, the average CAPRA-S scores did not differ statistically significantly between the nuclear HSF1 groups (*F* test *p* = 0.221) demonstrating non-dependency between HSF1 and CAPRA-S. Missing values in the data set were handled by multiple imputation, however no HSF1 data was imputed. 100 imputed datasets were created with varying imputed values reflecting the uncertainty of the imputation. The Cox model was fitted on each of the imputed datasets and collected into a single analysis. The variation between the imputed datasets was significantly smaller than the average estimation uncertainty for disease-specific survival (Supplementary Table 4). From these data, confidence intervals for the HRs were constructed using Rubin's rules.

All statistical analyses were performed with the programming language R, version 3.2 (R Foundation for Statistical Computing, http://www.R-project.org). Nonstandard external R-packages used include glmnet [53], ggplot2 [54], mi [55], riskRegression (https://CRAN.R-project.org/package=riskRegression) and rpart (https://CRAN.R-project.org/package=rpart).

## SUPPLEMENTARY MATERIALS FIGURES AND TABLES



## Abbreviations

BCR: biochemical recurrence
CAPRA-S: Cancer of the Prostate Risk Assessment post-Surgical
GG: grade group
GL: Gleason grade
HSF1: heat shock factor 1
HR: hazard ratio
IHC: immunohistochemistry
LASSO: least absolute shrinkage and selection operator
PSA: prostate-specific antigen
TMA: tissue microarray.
