# Supplementary material for: Increased HSF1 expression predicts shorter disease-specific survival of prostate cancer patients following radical prostatectomy
Source: Oncotarget. 2018 Jul 27;9(58):31200–13. doi: 10.18632/oncotarget.25756 (PMC6101287; doi:10.18632/oncotarget.25756)
Supplement: Supplementary file 1 [file oncotarget-09-31200-s001.pdf]

Increased HSF1 expression predicts shorter disease-specific survival of prostate cancer patients following radical prostatectomy

SUPPLEMENTARY MATERIALS

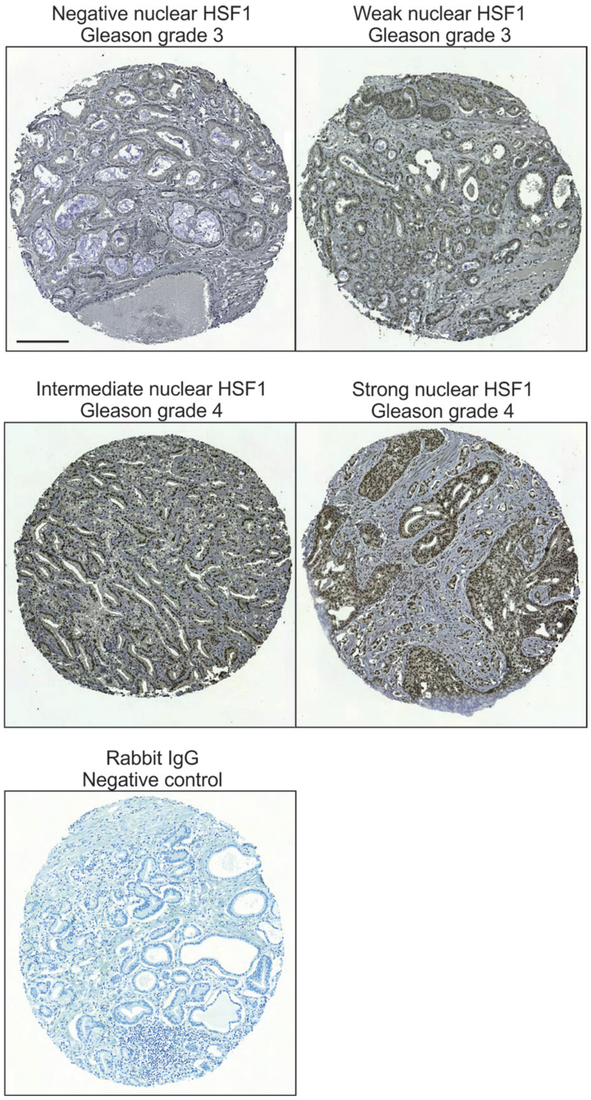

Supplementary Figure 1: HSF1 nuclear expression in TMA I. Representative cores with IHC staining of HSF1 showing negative (score 1), weak (score 2), intermediate (score 3), and strong (score 4) HSF1 protein expression. As a negative antibody control rabbit IgG staining was used. Scale bar represents 200 µm.

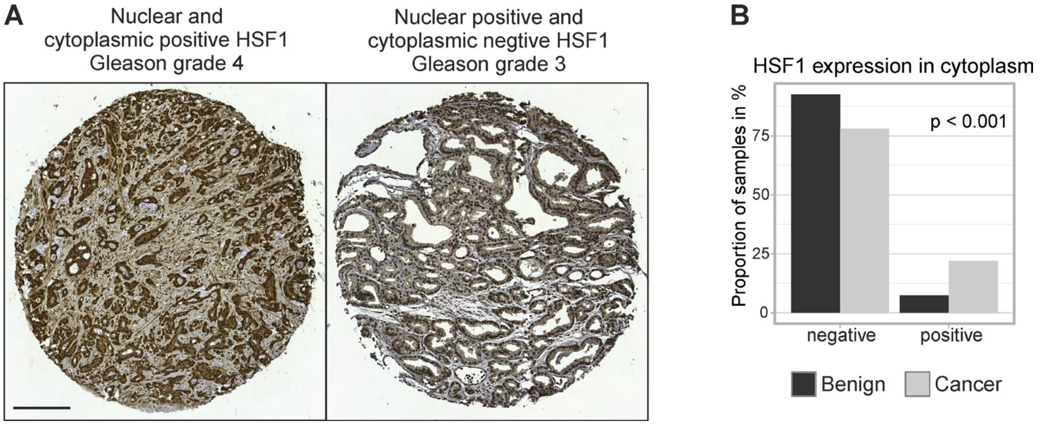

Supplementary Figure 2: HSF1 cytoplasmic expression. (**A**) IHC staining from TMA I, showing representative cores with both nuclear and cytoplasmic positive HSF1 expression (left) and positive nuclear but negative cytoplasmic HSF1 expression (right). Scale bar represents 200 µm. (**B**) Summary of the cytoplasmic HSF1 protein staining status (positive and negative) in benign/normal and malignant prostate cancer biopsies.

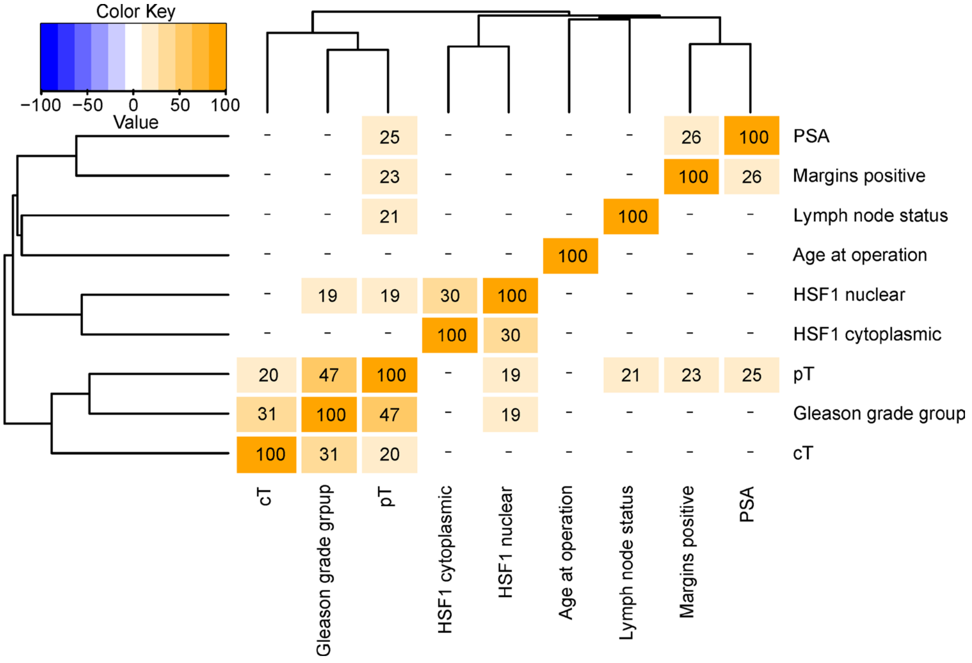

	cT	Gleason grade grpup	pT	HSF1 cytoplasmic	HSF1 nuclear	Age at operation	Lymph node status	Margins positive	PSA
PSA	-	-	25	-	-	-	-	26	100
Margins positive	-	-	23	-	-	-	-	100	26
Lymph node status	-	-	21	-	-	-	100	-	-
Age at operation	-	-	-	-	-	100	-	-	-
HSF1 nuclear	-	19	19	30	100	-	-	-	-
HSF1 cytoplasmic	-	-	-	100	30	-	-	-	-
pT	20	47	100	-	19	-	21	23	25
Gleason grade group	31	100	47	-	19	-	-	-	-
cT	100	31	20	-	-	-	-	-	-

Supplementary Figure 3: Clustered correlation heat map (pairwise Spearman) displaying dependencies between the clinical parameters in the primary sample data of TMA I. Statistical significance of the correlations was tested with a permutation test and corrected for type I error with the Bonferroni method. Correlations are reported as percent. Nonsignificant correlations ($p > 0.05$) were removed from the plot. cT, clinical tumor stage; PSA, preoperative PSA; pT, pathological tumor stage.

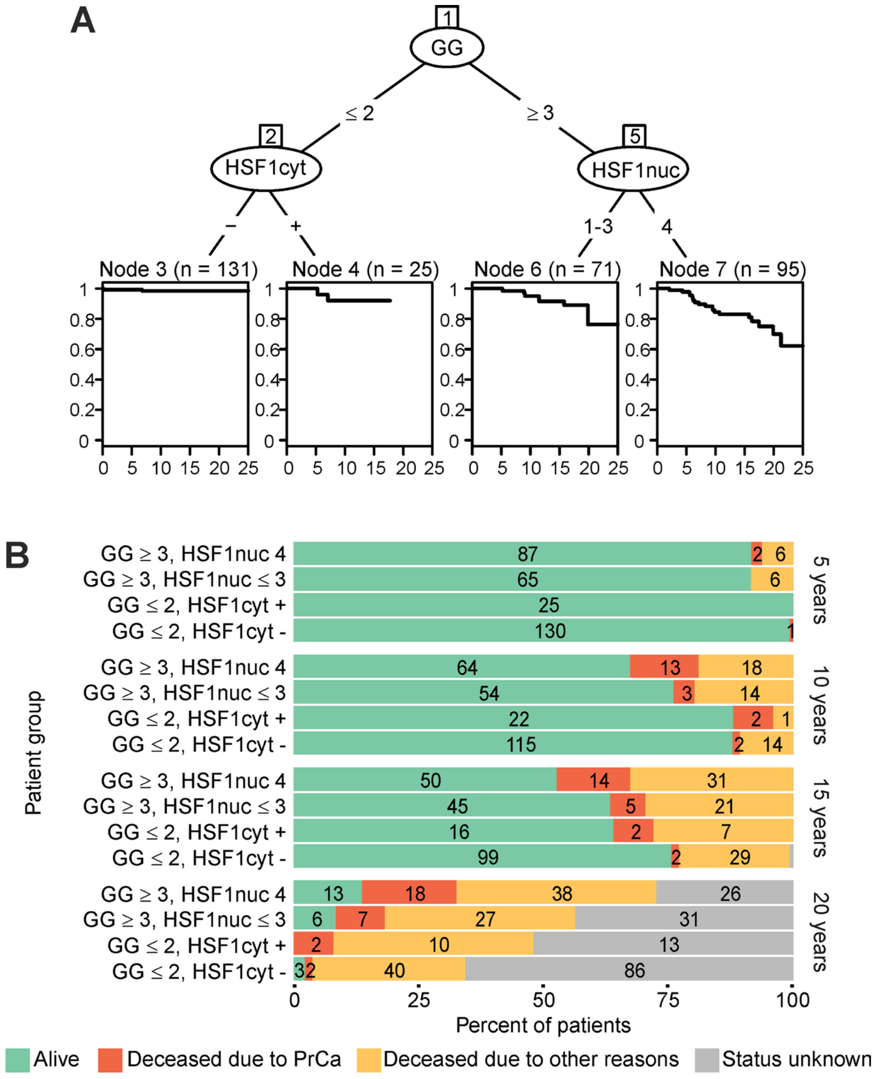

Supplementary Figure 4: Nuclear and cytoplasmic HSF1 expression status adds to disease-specific survival prediction together with grade group. (**A**) Survival tree. Covariates included are nuclear HSF1 expression (HSF1nuc, score 1–4 separately), cytoplasmic HSF1 expression (HSF1cyt) and grade group (GG). Node 1 distinguishes between GG ≤ 2 and ≥ 3, node 5 distinguishes between strong HSF1 expression (score 4) and negative, weak or intermediate HSF1 expression (score 1–3), node 2 distinguishes between negative (−) and positive (+) cytoplasmic HSF1 expression. (**B**) Survival status of patient groups corresponding to categories in A at fixed time points during follow-up. Values in the bars represent sample size. Green, patients alive at the time point; red, patients diseased due to cancer before the time point; yellow, patients diseased due to other reasons before the time point; gray, patients who did not yet reach the time point or were lost to follow-up.

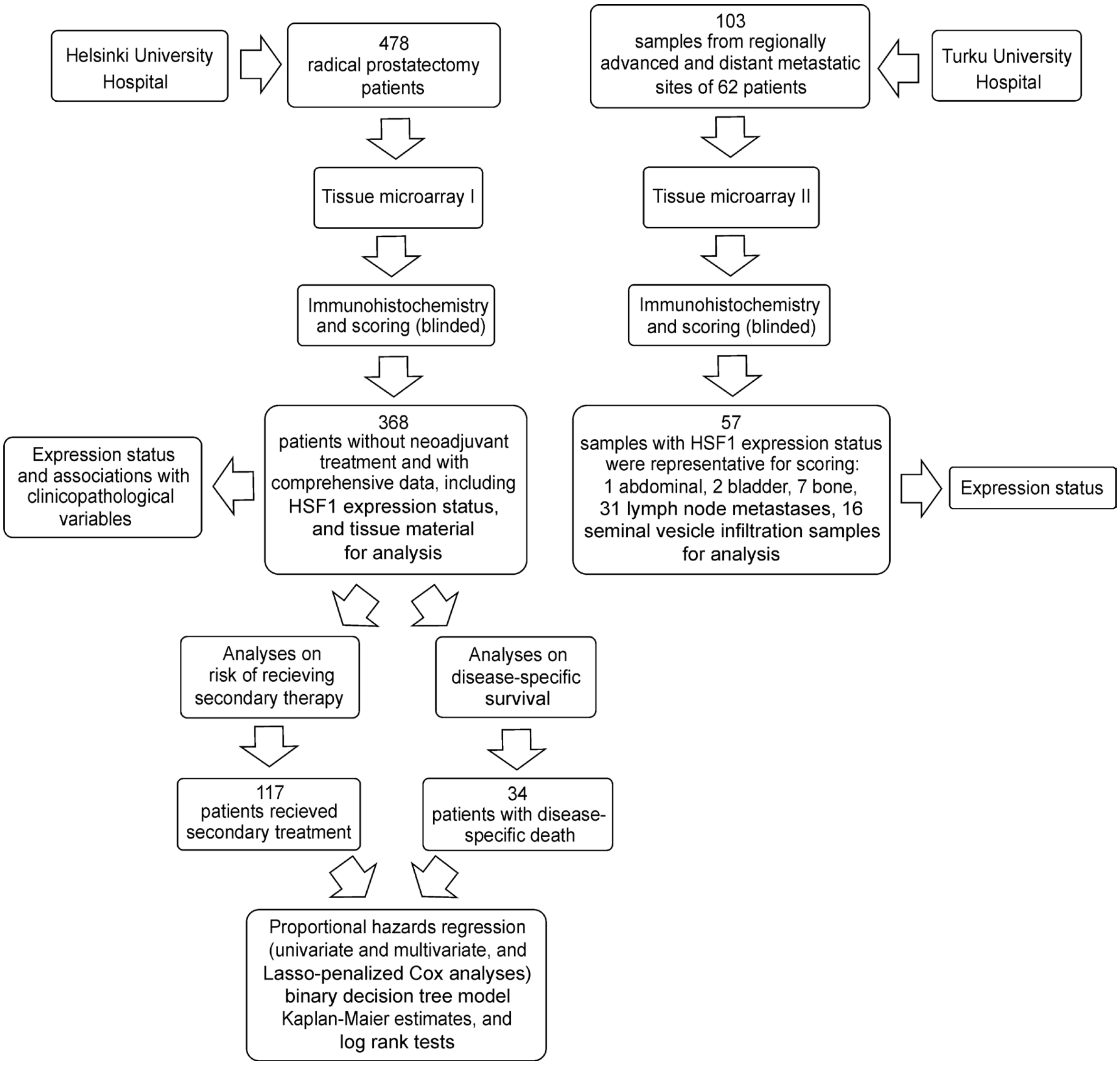

Supplementary Figure 5: Flow diagram on the study including number of patients and events at critical stages of the analyses following the reporting recommendations for tumor marker prognostic studies; REMARK [1].

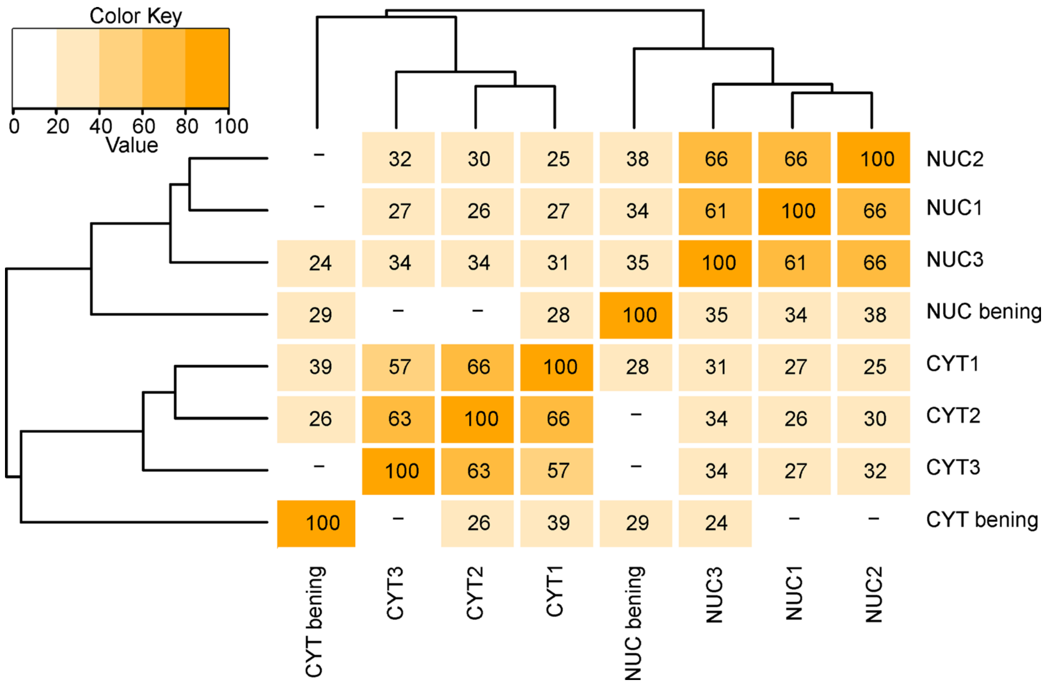

Supplementary Figure 6: Clustered correlation heat map (pairwise Spearman) displaying the correlation between HSF1 staining scores from the samples of TMA I. CYT1–3, cytoplasmic HSF1 expression from prostate cancer cores 1–3 from each patient; NUC1–3, nuclear HSF1 expression from prostate cancer cores 1–3 from each patient; CYT/NUC benign, cytoplasmic/nuclear HSF1 expression from the benign core. Statistical significance of the correlations was tested with a permutation test and corrected for type I error with the Bonferroni method. Correlations are reported as percent. Nonsignificant correlations ($p > 0.05$) were removed from the plot.

Supplementary Table 1: Demographics and clinicopathological variables of the prostate cancers in TMA I. 368 cancers with HSF1 expression status were analyzed from a total of 478 patients

Variables	Sample *n* (%)	Median (range)
**Age of patients at operation, years**	368	64 (45–76)
**Follow-up time, years**	335	15.7 (1.4–28.6)
**Preoperative PSA µg/l**	285	10.0 (0.0–120.0)
**Nuclear HSF1 expression status (maximum score)**		
Negative	14 (3.8)	
Weak	45 (12.2)	
Intermediate	115 (31.2)	
Strong	167 (45.4)	
Unknown	27 (7.3)	
**Cytoplasmic HSF1 expression (maximum score)**		
Positive	75 (20.4)	
Negative	267 (72.6)	
Unknown	26 (7.1)	
**Grade group** (Gleason score)		
**1** (4–6)	83 (22.6)	
**2** (3 + 4 = 7)	91 (24.7)	
**3** (4 + 3 = 7)	115 (31.3)	
**4** (8)	46 (12.5)	
**5** (9–10)	14 (3.8)	
Unknown	19 (5.2)	
**Tumor extension**		
pT2	193 (52.4)	
≥pT3	131 (35.6)	
Unknown	44 (12.0)	
**Positive surgical margins**		
Yes	62 (16.8)	
No	302 (82.1)	
Unknown	4 (1.1)	
**Lymph node status**		
Benign	353 (95.9)	
Metastasis	10 (2.7)	
Unknown	5 (1.4)	
**Secondary therapy after radical prostatectomy**		
Yes	117 (31.8)	
No	218 (59.2)	
Unknown	33 (9.0)	
**Patient outcome**		
Alive at the end of follow-up	189 (51.4)	
Deceased due to prostate cancer	34 (9.2)	
Deceased due to other reasons	143 (38.9)	
Status unknown	2 (0.5)	

Abbreviations: PSA, prostate-specific antigen.

Supplementary Table 2: Univariate and multivariate Cox proportional hazards models for risk of receiving secondary therapy in prostate cancers

**Univariate**	**Risk of secondary therapy**		
	**HR**	**95% CI**	***P***
HSF1 nuclear (score 4 *vs* 1–3)	1.56	1.03–2.35	0.035
HSF1 cytoplasmic (pos *vs* neg)	1.19	0.74–1.90	0.475
Grade group	2.03	1.70–2.42	<0.001
pT (≥T3 *vs* T2)	4.28	2.81–6.50	<0.001
PSA	1.03	1.02–1.04	<0.001
Positive surgical margin	2.50	1.65–3.81	<0.001
Lymph node metastasis	4.87	2.12–11.16	<0.001
**Multivariate[a]**	**HR**	**95% CI**	***P***
HSF1 nuclear (score 4 *vs* 1–3)	1.77	1.03–3.02	0.037
HSF1 cytoplasmic (pos *vs* neg)	0.5	0.24–1.03	0.06
Grade group	2.5	1.9–3.29	<0.001
pT (≥T3 *vs* T2)	2.1	1.22–3.59	0.007
PSA	1.02	1.01–1.03	0.002
Positive surgical margin	1.39	0.77–2.53	0.277
Lymph node metastasis	1.36	0.32–5.77	0.677

Abbreviations: CI = confidence interval; HR = hazard ratio.

[a]Number of patients, 237; number of events, 64.

Supplementary Table 3: Multivariate Cox proportional hazards models on disease-specific survival in prostate cancers

	**Disease-specific survival**		
	**HR**	**95% CI**	***P***
HSF1 nuclear (score 4 *vs* 1–3)	0.76	0.22–2.63	0.670
HSF1 cytoplasmic (pos *vs* neg)	1.53	0.36–6.55	0.566
Grade group	1.84	1.00–3.37	0.048
pT (≥T3 *vs* T2)	5.65	1.12–28.56	0.036
PSA	1.72	0.79–3.77	0.172
Positive surgical margin	0.42	0.10–1.80	0.232
Lymph node metastasis	2.3	0.23–23.53	0.482

Abbreviations: CI = confidence interval; HR = hazard ratio.
Number of patients, 245; number of events, 12.

Supplementary Table 4: Variation due to random noise and multiple imputation

	Disease-specific survival	
	**CAPRA-S**	**HSF1 nuclear**
Average estimate	0.2413	0.8208
Average STD of the estimate	0.0643	0.3988
STD between imputed datasets	0.0253	0.0345
Total STD	0.0691	0.4003

Abbreviations: STD, standard deviation.

REFERENCES

1. McShane LM, Altman DG, Sauerbrei W, Taube SE, Gion M, Clark GM, Statistics Subcommittee of the NCI-EORTC Working Group on Cancer Diagnostics. REporting recommendations for tumour MARKer prognostic studies (REMARK). Br J Cancer. 2005; 93:387–391.
